# Patient engagement in preclinical laboratory research: A scoping review

**DOI:** 10.1016/j.ebiom.2021.103484

**Published:** 2021-07-17

**Authors:** Grace Fox, Dean A. Fergusson, Zeinab Daham, Mark Youssef, Madison Foster, Evelyn Poole, Ayni Sharif, Dawn P. Richards, Kathryn Hendrick, Asher A. Mendelson, Kimberly F. Macala, Zarah Monfaredi, Joshua Montroy, Kirsten M. Fiest, Justin Presseau, Manoj M. Lalu

**Affiliations:** a Clinical Epidemiology Program, Blueprint Translational Research Group, Ottawa Hospital Research Institute, 501 Smyth Road, PO Box 201B, Ottawa, Ontario K1H 8L6, Canada; b School of Epidemiology and Public Health, University of Ottawa, Canada; c Ottawa Stroke Program, Ottawa Hospital Research Institute, Canada; d Faculty of Medicine, University of Ottawa, Canada; e Patient Partner, Five02 Labs Incorporated, Canada; f Patient Partner, Toronto, Ontario, Canada; g Section of Critical Care Medicine, Department of Medicine, Rady Faculty of Health Sciences, University of Manitoba, Canada; h Department of Critical Care Medicine, Royal Alexandra Hospital, University of Alberta, Canada; i Department of Anaesthesiology and Pain Medicine, Royal Alexandra Hospital, University of Alberta, Canada; j Faculty of Health Sciences, Simon Fraser University, Canada; k Department of Critical Care Medicine, Cumming School of Medicine, University of Calgary & Alberta Health Services, Canada; l School of Psychology, University of Ottawa, Canada; m Department of Anaesthesiology and Pain Medicine, The Ottawa Hospital, Canada

**Keywords:** Patient engagement, Preclinical, Basic science, Translational research, Scoping review

## Abstract

**Background:**

‘Patient engagement’ involves meaningful collaboration between researchers and ‘patient partners’ to co-create research. It helps ensure that research being conducted is relevant to its ultimate end-users. Although patient engagement within clinical research has been well documented, the prevalence and effects of patient engagement in translational preclinical laboratory research remain unclear. The aim of this scoping review is to present current patient engagement activities reported in preclinical laboratory research.

**Methods:**

MEDLINE, Embase, and grey literature were systematically searched from inception to April 2021. Studies that described or investigated patient engagement in preclinical laboratory research were included. Patient engagement activities where patients (i.e. patients, family members, caregivers or community members) provided input, or consultation on at least one element of the research process were eligible for inclusion. Study characteristics and outcomes were extracted and organized thematically.

**Findings:**

32 reports were included (30 primary studies, 1 narrative review, and 1 researcher guide). Most studies engaged patients at the education or priority setting stages (*n*=26). The most frequently reported benefit of patient engagement was ‘providing a mutual learning opportunity’. Reported barriers to patient engagement reflected concerns around ‘differences in knowledge and research experience’ and how this may challenge communication and limit meaningful collaboration.

**Interpretation:**

Patient engagement is feasible and beneficial for preclinical laboratory research. Future work should focus on assessing the impacts of patient engagement in this area of research.

**Research in context**

**Evidence before this study**

Patient engagement in clinical research is well established and has been observed to enhance research in various ways including increased study relevance, improved trial recruitment and retention. Funding agencies, top-tier journals, and other stakeholders have recognized patient engagement as an important and necessary facet of research. Despite this, the extent and impact of patient engagement in preclinical laboratory research is unclear.

**Added value of this study**

To our knowledge, this is the first scoping review to capture current patient engagement practices in preclinical research. Our review captured 32 articles that describe or study patient engagement at various stages (e.g. research priority setting, funding, study design, dissemination of findings) of preclinical laboratory research. Key reported benefits of patient engagement included mutual learning opportunities, establishing new collaborations, and improved research efficiency.

**Implications of all the available evidence**

These findings present the current landscape of patient engagement in preclinical research and identified barriers and enablers to engagement in this field. Despite the paucity of published evidence, our results demonstrate that engaging patients in preclinical research is feasible and may enhance research conduct in unique ways. Our results should encourage preclinical researchers and patient partners to establish new collaborations.

Alt-text: Unlabelled box

## 1 Introduction

The movement to involve patients in scientific research, also known as patient engagement, refers to meaningful collaboration between researchers and patient partners [1]. The term *patient* encompasses individuals with lived experience of a health issue and informal caregivers, including family and friends [2]. In particular, patients are actively engaged throughout research development and conduct (e.g. governance, developing the research questions or even performing certain parts of the research itself) rather than being participants of research [1]. Several benefits of patient engagement in clinical research have been postulated, including: aligning research with patient priorities [3]; improving content and documentation [3]; increasing participant recruitment in clinical trials [4]; and enhancing the accessibility and dissemination of findings to the public [5]. Many stakeholders and policymakers suggest that underlying ethical, moral, and political arguments justify such engagement activities, since patients are the ultimate end-users of medical research and therefore should be involved in its production [3,5].

Considering that most published evidence of patient engagement comes from clinical research, the current standing of patient engagement in preclinical research (i.e. laboratory research conducted in cell and animal models) is unclear. This may be particularly germane to the ultimate translation of preclinical laboratory-based discoveries, since partnering with patients at this early stage of research could help align preclinical research with patient priorities. It is also important as the majority of health research funding is directed to fundamental and preclinical research [6]. The extent to which patients are currently involved in preclinical laboratory research as collaborators (i.e. patient partners) is unestablished, however this likely reflects several issues. First preclinical laboratory research is not a traditional patient-facing domain of biomedicine and preclinical scientists do not typically interact with patients (unlike clinical researchers). Thus, preclinical laboratory research may be considered removed from patient priorities and interests. Second, although infrastructure exists for patient engagement in clinical research, similar resources for preclinical patient engagement are not yet widespread. Finally, several key issues are currently unclear, including the prevalence and effects of patient engagement, and what mechanisms may effectuate engagement.

Investigating how preclinical researchers have implemented patient engagement and overcome barriers is paramount to understanding effective patient engagement in preclinical laboratory research. We performed a scoping review to examine the current landscape of patient engagement in preclinical laboratory research and identify current trends in this setting.

## 2 Methods

We followed a standard scoping review framework, first defined by Arksey and O'Malley [7], then later expanded by Levac *et al.*[8], and the Joanna Briggs Institute [9]. This review is reported in accordance with the PRISMA extension for scoping reviews (a completed checklist can be found in Appendix 1) [10]. The protocol and updates for this review can be found in Open Science Framework (osf.io/qf5z7). A completed Guidance for Reporting Involvement of Patients and the Public (GRIPP2) checklist can be found in Appendix 2 [11].

### 2.1 Information sources and literature search

A qualified information specialist systematically searched Embase and MEDLINE (from inception of each database, the search was last updated on April 6, 2021). No date restrictions were applied to be as comprehensive as possible in our search. Keywords related to basic science, research, laboratory, biomedical, patient engagement, and public involvement were used. The full search strategy used can be found in Appendix 3. Original studies, case reports, reviews (narrative and systematic), and opinion piece articles that report on patient engagement in preclinical laboratory research were eligible. Abstracts were excluded as they lacked details required for our synthesis (e.g. detailed descriptions of patient engagement activities). Identified review articles underwent a backward citation check to identify any additional articles eligible to be included in the scoping review.

In addition, we conducted a grey literature search in accordance with the guidance published by the Canadian Agency for Drugs and Technologies for Health; the Grey Matters Checklist [12]. Three Google searches were conducted on March 23, 2020 using the following keywords selected in collaboration with an information specialist: “patient engagement” and “basic science”, “patient involvement” and “laboratory research”, and “patient involvement” and “biomedical research”. Similarly, the ProQuest Dissertations and Theses Global^TM^ repository of graduate dissertations and theses was searched using the following key terms: “patient engagement” and “basic science”. ProQuest is a North American repository. The first fifty articles from each search (200 articles in total) were collected for screening.

### 2.2 Eligibility criteria and study selection

Eligible sources described or studied patient engagement in preclinical laboratory research. We defined “preclinical laboratory research” as non-clinical research performed *in vitro* or *in vivo* (i.e. laboratory experiments conducted with cells or animals, respectively) that aims to increase understanding of a human condition [13]. In accordance with the Canadian Institutes for Health Research's Strategy for Patient Oriented Research (SPOR), we defined “patient engagement” broadly as any activity where patients, family members, caregivers, and community members provided input, guidance, or consultation on at least one element of the research process. Elements included awareness/education/training, topic generation, priority-setting, governance, question refinement, defining outcomes, methods and study design, statistical analysis plan, conduct of research, interpretation of results, and dissemination and implementation of results [1]. Priority-setting activities that involved preclinical scientists and preclinical research priorities were included. Survey and interview studies of patients identifying preclinical research priorities or directly informing study design were included. Similarly, articles written by or about patient organizations’ roles in governing policy development and funding decisions of preclinical research were included. Articles about or describing patient engagement in *clinical* research or clinical care were excluded.

Articles were uploaded to DistillerSR® (Evidence Partners, Ottawa, Canada), an audit-ready, cloud-based software program. Five team members were responsible for screening and data extraction (GF, MF, ZD, MY, AS). Two independent reviewers screened articles in duplicate by titles/abstracts and then by full text. A calibration exercise was performed on the first fifty articles to refine the screening question prior to formally commencing the screening process, and to ensure reviewers understood and interpreted the eligibility criteria correctly. Discrepancies between the reviewers were resolved by discussion or by a senior team member. Reasons for exclusion were recorded at the full-text phase.

### 2.3 Data extraction

Two reviewers abstracted data from included studies independently using a standardized data extraction form. Senior team members provided oversight throughout screening and data extraction (MML, JP, DR). The extraction form was developed with input from all team members and the GRIPP2 checklist [11]. A full list of items extracted can be found in Appendix 4. We used the International Association of Public Participation (IAP2) spectrum [14] to categorize the level of patient engagement as either: inform, consult, involve, collaborate, or empower. We added a sixth level (awareness) to capture examples of engagement at the education or advocacy level (Supplementary Fig. 2). The IAP2 spectrum aligns with work conducted by Carman et al. [15] to develop a framework that distinguishes between different levels of patient engagement in health care decision making. We categorized patient engagement as contributing to various stages of research within the research project (i.e. education, funding, priority setting, study design, data collection and analysis, dissemination of results, and awareness). To capture methods of crediting patient partners for their contribution, we categorized methods as financial compensation (e.g. stipend, honorarium), reimbursement (i.e. travel or accommodation expenses), gift (e.g. gift card in lieu of financial compensation), publication acknowledgement, and co-authorship.

### 2.4 Synthesis of results

Our content analysis followed a 6-step approach developed by Thomas and Harden [16] and later implemented by Ryan et al. [17]: (i) four independent reviewers (GF, ZD, MY, AS) extracted verbatim statements of benefits, challenges, recommendations, barriers, and enablers to patient engagement in preclinical laboratory research from each included study. Any discrepancies in extraction were resolved by reviewers with the help of a senior author if consensus could not be reached (MML); (ii) statements were presented to the entire research team and analyzed within each of the 5 domains; (iii) two reviewers (EP, GF) categorized statements as reoccurring themes. Reoccurring themes were grouped together, and new themes were identified as they emerged. This was achieved through an analytic framework that organized overarching themes as table columns and verbatim statements in the rows of each corresponding theme; (iv) categorization was reviewed by a second reviewer (GF, MF, AS). The tabulated results were presented to the entire research team for review and refinement; (v) results were narratively synthesized by two reviewers (GF, MML) and overarching themes were presented as tables; (vi) overarching themes were ordered by frequency within each patient engagement domain.

### 2.5 Patient partner engagement

Our patient partners were recruited through their involvement in past projects and personal referrals. Our patient partners were involved in the initial grant applications as co-applicants, helped co-develop the research question, participated in bi-monthly meetings, provided input on data extraction items, and helped synthesize the results of this study. Both patient partners are co-authors. Further details about engagement activities and our patient partners can be found in Appendix 2.

### 2.6 Ethics

Ethical approval was not required.

### 2.7 Role of funding source

This work was supported by Canadian Institutes of Health Research (CIHR) Strategy for Patient Oriented Research Catalyst Grant: Patient-Oriented Research and a CIHR Project Grant. MML is supported by The Ottawa Hospital Anesthesia Alternate Funds Association and holds a University of Ottawa Junior Research Chair in Innovative Translational Research. The funders had no role in the study design; in the collection, analysis, and interpretation of data; in the writing of the report; and in the decision to submit the article for publication. All researchers are independent from funders and all authors, external and internal, had full access to all of the data in the study (including statistical reports and tables) and take responsibility for the integrity of the data and the accuracy of the data analysis.

## 3 Results

### 3.1 Search and study characteristics

**Fig. 1:**
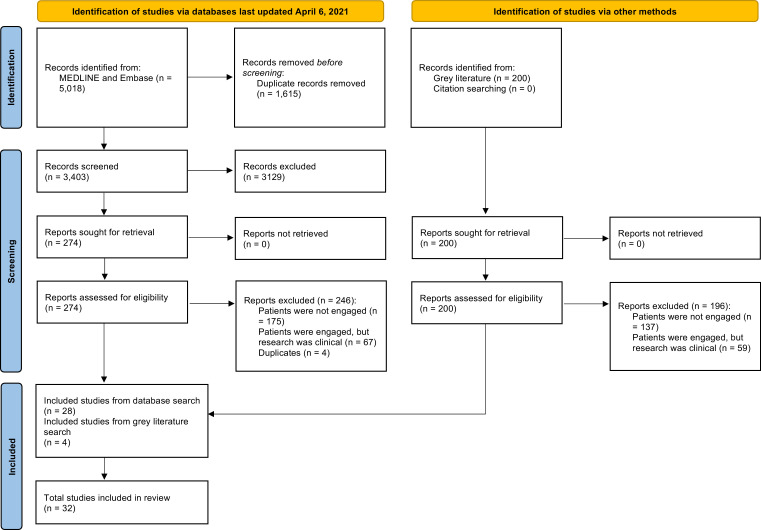
PRISMA flow diagram.

A total of 3603 articles were screened. Thirty-two articles met eligibility criteria, including 30 articles that described 29 primary studies, one narrative review, and one researcher guide. This is represented in the PRISMA flow diagram (Fig. 1). Study characteristics were extracted from primary studies only, and all 32 articles were included in the content analysis (Fig. 1). Of note, the narrative review included had synthesized eight recommendations for including patients in scientific research through a literature search of preclinical and clinical research.

Included articles were published between 2007 and 2021 (Table 1). Studies were conducted predominantly in the United Kingdom (*n*=12) and the United States (*n*=9) (Supplementary Fig. 1). A variety of disease domains were investigated with the most common being genetic (*n*=8) and musculoskeletal (*n*=7) conditions. Studies pertaining to *in vitro* (*n*=9), *in vivo* (*n*=5), and both *in vitro* and *in vivo* (*n*=5) methods were identified, while the remaining studies did not specify the type of research under investigation (*n*=12).

**Table 1:** Study characteristics of included articles (*n*=32 articles).

Author, Year	Country	Area of research	Type of research	Type of funding	Patient engagement specific funding
Rheault et al. [18]	United Kingdom	Alport syndrome	*In vitro, In vivo*	A, F	N/R
van den Berg et al. [19]	Netherlands	Amyotrophic lateral sclerosis (ALS)	*In vitro, In vivo*	G, F, I	N/R
Boenink et al. [20]	Netherlands	Advanced stage cancer and rheumatoid arthritis	*In vitro*	G	Explicit statement of funding received to support patient engagement
Russell et al. [21]	United Kingdom	Autism	*In vivo*	G, F, I	N/R
Tamagnini et al. [22]	United Kingdom	Alzheimer's and dementia	*In vivo*, Ethics of animal research	F	Explicit statement of funding received to support patient engagement
Frazier et al. [23]	United States	Autism	N/R	N/R	
Talebizadeh et al. [24]	United States	Genetics	N/R	G	No explicit statement, but the grant name suggests it may be to support patient engagement
McDonnell et al. [25]	United Kingdom	Lupus and antiphospholipid syndrome	N/R	G, A, F	N/R
Parsons et al. [26]	United Kingdom	Various arthritis diseases and bone disease	N/R	A, F	No explicit statement, but the grant name suggests it may be to support patient engagement
Zoeller [27]	Germany	Osteoarthritis	*In vivo*	N/R	
Filocamo et al. [28]	Italy	Rare diseases	*In vitro*	G, F	N/R
Black and Brockway-Lunardi [29]	United States	Melanoma	*In vitro, In vivo*	N/R	
Godard et al. [30]	Canada	Genetics	N/R	G	N/R
Author, Year	Country	Area of research	Type of research	Type of funding	Patient engagement specific funding
*Haga et al. [31]	United States	Genetics	*In vitro*	G	N/R
*O'Daniel et al. [32]	United States	Genetics	*In vitro*	G	No explicit statement, but the grant name suggests it may be to support patient engagement
Terry et al. [33]	United States	Genetics	*In vitro*	A	Explicit statement of funding received to support patient engagement
Pulver et al. [34]	United Kingdom	Animal models for biomedical research	*In vivo*	G, F	N/R
Arturi [35]	United States	Diamond Blackfan Anemia	*In vitro*	N/R	
Baart and Abma [36]	Netherlands	Psychiatric genomics	N/R	N/R	
Boon and Broekgaarden [37]	Netherlands	Neuromuscular disorders	*In vitro, In vivo*	N/R	
Van Olphen et al.[38]	United States	Breast cancer	*In vivo*	G	Explicit statement of funding received to support patient engagement
Haddow et al. [39]	United Kingdom	Genetics	*In vitro*	N/R	
Riter and Weiss [40]	United States	Cancer	N/R	G, A	N/R
de Wit et al. [41]	Netherlands	Rheumatic conditions	N/R	N/R	
Mollan et al. [42]	United Kingdom	Idiopathic intracranial hypertension	N/R	G, F	Explicit statement of funding received to support patient engagement
Costello and Dorris [43]	Ireland	Rheumatic conditions	*In vitro, In vivo*	N/R	
Arthritis Research UK, N/R [44]	United Kingdom	Rheumatic conditions	N/R	N/R	
Author, Year	Country	Area of research	Type of research	Type of funding	Patient engagement specific funding
Davies et al. [45]	United Kingdom	Genetics	*In vivo*	F	No explicit statement, but the grant name suggests it may be to support patient engagement
Taruscio et al. [46]	Italy	Rare diseases	N/R	N/R	
Moore et al. [47]	United Kingdom	Multiple sclerosis	*In vitro*	F	N/R
Mahler and Besser [48]	Germany	Stem cells	*In vitro*	N/R	
Birch et al. [49]	United Kingdom	Rheumatoid arthritis	N/R	G, A, F	N/R

Abbreviations: A = Academic, F = Foundation/Charity, G = Government, I = Industry, N/R = Not reported

⁎ Denotes articles describing the same study.

### 3.2 Patient engagement characteristics and identification

Across the 30 included primary studies, a variety of patient partners were engaged including patients (*n*=20), community members (*n*=16), members of patient organizations (*n*=11), family members (*n*=11), caregivers (*n*=7), and friends (*n*=1) (Table 2a). Nineteen studies reported engaging more than one type of patient stakeholder. Nineteen studies reported the number of engaged patients with a median of 47 (3-4885) (Table 2a). The study engaging the largest number of patients did so through a priority setting questionnaire [23]. Fourteen studies reported on at least one demographic feature of patient partners (i.e. age, gender, language, ethnicity, education level, profession) (Supplementary Table 1), but inconsistent reporting limited our ability to assess diversity of patient partners. Twenty-three studies reported a method of recruitment with the most common method being partnership with a patient organization (n=15) (Table 2b). One study recruited patient partners to the Governing Board through an application process that required a referral letter from a primary licensed healthcare provider [46].

**Table 2a:** Patient engagement characteristics of included studies (n=30).

Study details	Type of stakeholder engaged						Number of patient partners engaged	Type of engagement (duration)
	Patients	Community members	Members of patient organizations	Family members	Caregivers	Friends		
Rheault et al. [18]	√		√	√			N/R	Pre-conference and conference events (NR)
van den Berg et al. [19]	√	√	√		√		N/R	Face-to-face workshop (2-days)
Boenink et al. [20]	√						N/R	One-time engagement (N/R)
Russell et al. [21]	√	√		√	√		66	2 events (1-year apart) with email follow-up (N/R)
Tamagnini et al. [22]		√		√	√		3	N/R
Frazier et al. [23]	√		√	√	√		4885	One-time engagement (N/R)
Talebizadeh et al. [24]	√	√		√			12	Attendance at 6 sessions over a 12-month period
McDonnell et al. [25]	√						523	One-time engagement (N/R)
Parsons et al. [26]	√						63	One focus group (90 min)
Zoeller [27]	√	√					71	Attendance at meetings (Two weekends)
Filocamo [28]			√				N/R	Attendance at several meetings and workshops (N/R)
Black and Brockway-Lunardi [29]	√		√				N/R	N/R
Godard et al. [30]		√					1,568	One-time engagement (N/R)
*Haga et al. [31]		√					159	8 group sessions (N/R)
*O'Daniel et al. [32]		√					159	8 group sessions (N/R)
Terry et al. [33]		√					N/R	2-year project
Pulver et al. [34]		√					53	One-time engagement (N/R)
Arturi [35]	√	√					N/R	N/R
Baart and Abma [36]	√			√			16	1-year project
Boon and Broekgaarden [37]			√	√			N/R	N/R
Van Olphen et al. [38]	√	√	√				9	N/R
Haddow et al. [39]	√	√	√				N/R	N/R
Riter and Weiss [40]	√	√	√	√	√		12+	N/R
Mollan et al. [42]	√			√	√	√	122	18-month project
Costello and Dorris [43]	√		√	√	√		41	Attendance at a conference and a workshop (N/R)
Davies et al. [45]		√					N/R	Attendance at three workshops and completion of a survey (N/R)
Taruscio et al. [46]	√		√				3	Governing Board member (3 years)
Moore et al. [47]	√	√					4	Attendance at four meetings (6 h)
Mahler and Besser [48]		√					N/R	N/R
Birch et al. [49]	√			√			9	4-year project
Total (%)⁎⁎	20 (69)	16 (55)	11 (38)	11 (38)	7 (24)	1 (3)		

⁎ Denotes articles describing the same study.

⁎⁎ Percentages were generated using n=29 as the denominator.

**Table 2b:** Patient engagement characteristics of included studies (*n*=30).

Study details	Method of stakeholder recruitment						Stage of research where patient partners contributed							
	Partnering with other organization	Social marketing	Other	Community outreach	Health system	N/R	Education	Funding	Priority setting	Study design	Data collection	Data analysis	Dissemination of results	Awareness
Rheault et al. [18]	√								√					
van den Berg et al.[19]						√			√	√				
Boenink et al.[20]						√			√	√				
Russell et al.[21]	√	√					√		√					√
Tamagnini et al.[22]	√√						√		√	√				√
Frazier et al.[23]	√	√							√					
Talebizadeh et al.[24]			√			√			√	√				
McDonnell et al.[25]	√	√							√	√				
Parsons et al.[26]	√				√				√					
Zoeller [27]						√			√				√	
Filocamo et al.[28]						√	√		√√	√			√	√
Black and Brockway-Lunardi[29]	√						√	√	√					√
Godard et al. [30]	√												√	
*Haga et al.[31]		√					√						√	√
*O'Daniel et al.[32]		√					√							
Terry et al.[33]	√	√					√		√	√			√	
Pulver et al.[34]				√			√							
Arturi [35]			√				√	√	√				√	√
Baart and Abma [36]	√						√		√					√
Boon and Broekgaarden [37]						√	√	√	√					√
Van Olphen et al. [38]	√						√	√					√	
Haddow et al.[39]						√	√							
Riter and Weiss[40]	√	√					√							
Mollan et al. [42]		√								√				
Costello and Dorris[43]	√												√	
Davies et al. [45]						√	√		√					
Taruscio et al.[46]			√				√		√					
Moore et al.[47]	√						√			√			√	
Mahler and Besser[48]				√			√							
Birch et al. [49]	√		√										√	√
Total (%)⁎⁎	15 (52)	7 (24)	4 (14)	2 (7)	1 (3)	8 (28)	17 (59)	4 (14)	18 (62)	9 (31)	0 (0)	0 (0)	10 (34)	9 (31)

Study details	Level of engagement													
	Awareness/Education	Inform	Consult	Involve	Collaborate	Empower								
Rheault et al. [18]				√	√									
van den Berg et al.[19]			√		√									
Boenink et al.[20]				√	√									
Russell et al.[21]	√	√	√	√										
Tamagnini et al.[22]	√	√	√	√	√									
Frazier et al.[23]			√											
Talebizadeh et al.[24]			√											
McDonnell et al.[25]		√	√	√										
Parsons et al.[26]				√	√									
Zoeller [27]					√									
Filocamo et al.[28]					√									
Black and Brockway-Lunardi[29]	√					√								
Godard et al. [30]			√		√									
*Haga et al.[31]					√									
*O'Daniel et al.[32]					√									
Terry et al.[33]	√				√	√								
Pulver et al.[34]				√										
Arturi [35]	√				√	√								
Baart and Abma [36]	√	√	√											
Boon and Broekgaarden [37]						√								
Van Olphen et al. [38]	√													
Haddow et al.[39]					√									
Riter and Weiss[40]	√	√	√											
Mollan et al. [42]			√	√		√								
Costello and Dorris[43]	√	√	√			√								
Davies et al. [45]			√	√										
Taruscio et al.[46]				√										
Moore et al.[47]		√			√									
Mahler and Besser[48]		√												
Birch et al. [49]					√									
Total (%)⁎⁎	9 (31)	8 (28)	12 (41)	10 (34)	14 (48)	6 (21)								

Methods of stakeholder recruitment were categorized using the following criteria:

Community outreach: town hall meetings with community leaders or visiting schools

Health system: health care providers

Patient organizations: advocacy groups or charitable organizations

Other: personal or professional referrals

Social marketing: advertisements on radio, TV, newspapers, social media, and public spaces such as churches, schools, libraries and waiting rooms

Abbreviations: N/R = Not reported

⁎ Denotes articles describing the same study.

⁎⁎ Percentages were generated using n=29 as the denominator.

### 3.3 Patient engagement activities

The majority of primary studies engaged patients at the education (*n*=17) or priority setting stages (*n*=18) of the research project (Table 2b), where activities included information sharing and round table discussions, respectively. Notably, five included studies described patient partner contribution in planning and conducting priority setting focus groups or surveys and subsequent transcription and analysis of discussions.

Levels of engagement (awareness, inform, consult, involve, collaborate, empower) are not mutually exclusive and patient partners can be engaged at multiple levels within one research project. We captured six studies that engaged patient partners at the empowerment level where patients are the ultimate decision makers (Table 2b). Patient partners held positions on committees in three of these studies whereby they provided direction to the research project. Two studies were written by patient founded organizations outlining funding decisions for preclinical research.

### 3.4 Researcher and patient partner training

Three studies reported offering training sessions for researchers to facilitate patient engagement, which included exercises to improve communicating research to non-scientists. One study reported offering training to patient partners on specific research activities.

### 3.5 Funding and credit for patient engagement

Five studies explicitly stated that they received funding to support patient engagement (Table 1). An additional four studies reported receiving funding from agencies known to support patient engagement initiatives. Two studies commented on the cost of patient engagement and both noted that overall costs were minimal.

Two studies reported financially compensating their patient partners, two studies reimbursed travel expenses, and one study offered a gift card in place of monetary compensation, while 23 studies did not report on financial compensation of patient partners (Supplementary Table 2). Credit for engagement was mostly provided through acknowledgement of contribution within the manuscript (*n*=11) (Supplementary Table 2). One study listed their patient partner as a co-investigator on the research project [38].

### 3.6 Benefits and challenges of patient engagement

Five themes emerged from our analysis of reported benefits of patient engagement (Table 3). The most frequently reported benefits reflect the two themes of ‘mutual learning opportunities’ and the ‘opportunity to build new skills, knowledge, and interest’. Engagement increased patient partners’ understanding of basic science research, while preclinical researchers had their perspective broadened including a better understanding of patient priorities. The third identified theme focused on patient partner input ‘improving study quality and efficiency’. Specifically, dialogue fostered by patient engagement directly informed study questions and methods, and improved dissemination of final results. The fourth theme centred on ‘improving communication to the public and strengthening research through trust’ as patient engagement generated stronger bonds between patient and research communities. Interestingly, for patient partners this also resulted in greater reported self-confidence and a sense of finding their ‘voice’. A final theme was an ‘increase in trainee recruitment/retention, external collaboration, and recruitment’. Trainees reported renewed motivation for basic science research through better understanding real-life implications of their research. Patient partners also fostered *de novo* collaborations between research groups (especially for rare diseases) and helped improve recruitment of participants in studies (e.g. to provide biological samples).

**Table 3:** Reported benefits and challenges of patient engagement (*n*=32).

Benefits	Studies
**A mutual learning opportunity [R/P]**	16
Patient engagement facilitates patient partner understanding and interest in basic science research	
Patient partners can improve researcher understanding of the real-life priorities and impact of their work	
**An opportunity to build new skills, knowledge, interests, and perspectives [R/P]**	13
Engagement experiences can inform and broaden perspectives of researchers	
Engaging a diverse patient partner group provides a greater understanding of diverse experiences	
**Patient partner input can improve study quality and efficiency [R/P]**	9
Patient engagement informs the research question, study methodology, and future research by fostering important discussions	
Patient partners can play an important role in disseminating research findings	
**Improves communication with the public and strengthens the research through trust [R/P]**	8
Encourages a sense of partnership (between patients and researchers) and improves patient partner trust of the research community	
Increases self-confidence and the impact of the patient voice	
**May increase trainee recruitment/retention, external collaboration, and recruitment [R]**	5
There is potential to create external partnerships that are rare for professional engagements	
Improved trainee retention by renewing interest in the real-life implications of their research	
Challenges	Studies

**Differences in research knowledge and experience between research and patient partner populations might affect the quality of research and limit meaningful engagement [R/P]**	3
Patient partners have difficulties connecting with future innovations	
**Engaging few patient partners can limit diverse perspectives**	1

Abbreviations: R and P denote themes that pertain to researchers and patient partners, respectively.

Two themes emerged from our analysis of reported challenges of patient engagement (Table 3). The first theme highlighted ‘differences in knowledge and experience between researchers and patient partners affecting the quality of research and limits meaningful engagement’ as a perceived challenge. The second theme acknowledges challenges associated with engaging a small sample of patient partners and how this may limit perspectives brought to a research project. Conversely, it was suggested engaging multiple patient partners may help ensure diverse perspectives are considered.

### 3.7 Recommendations for patient engagement

Reported lessons learned from the engagement activities of included articles were extracted to synthesize a list of recommendations to guide basic scientists in engaging patient partners in research. Our thematic analysis generated ten overarching themes (Supplementary Table 3). The most commonly reported theme recommended researchers ‘aim to recruit a diverse group of patient partners’. This theme echoes a reported challenge of engagement as it was difficult to recruit diverse groups of patient partners to ensure consideration of many unique perspectives.

Attaining diversity can be achieved through ‘collaborating with a patient organization to reach and recruit a diverse population’. Similarly, another theme supported ‘partnerships with patient organizations’ as this relationship could ‘help dissolve barriers between patient partners and preclinical researchers’. Three themes outlined lessons to consider before engaging patient partners. These included ‘providing educational resources to team members’ covering sufficient background information and rationale for the project, planning to ‘recognize patient partner contributions’ through compensation or acknowledgement for example, and the importance of ‘timing patient engagement’. Ideally, patients would be engaged from study onset to completion. Four themes outline items to consider throughout a patient engagement activity including ‘effective and consistent communication’ (e.g. roles, responsibilities, and expectations), acknowledging that ‘patient partners may become engaged to different degrees’ depending on availability and skills, expecting ‘disagreements and frustrations’ to occur, and ‘considering the patient partner and researcher relationship as collaborative’. The final recommendation was one to consider at the end of the research project and that is how pivotal ‘evaluating the impact of patient engagement’ is in ‘improving bi-directional knowledge translations’ and how this will affect the success of future initiatives.

### 3.8 Perceived barriers and enablers of patient engagement

Reported barriers to patient engagement in preclinical research were summarized into four themes (Table 4). Two of these themes highlighted infrastructure and resources such as ‘structural barriers’ like time and budget to support engagement, and the ‘lack of researcher and patient partner training opportunities to guide meaningful patient engagement in basic science research’. The other two themes emphasized group composition and dynamics. The ‘inadvertent exclusions of members of particular groups’ was a barrier to creating a representative patient partnership, and when a partnership was formed ‘addressing the priorities of all team members’ was a continuing issue.

**Table 4:** Reported barriers and enablers to patient engagement (*n*=32).

Barriers	Studies
**Addressing the priorities of all team members can be difficult to achieve [R]**	8
It is challenging to capture diverse viewpoints and research priorities from members with different research backgrounds	
**Structural barriers to patient engagement exist [R]**	7
Insufficient researcher resources to support patient partners including time and budget restrictions	
**Defining the patient partner population to recruit can be challenging [R]**	5
Recruitment may inadvertently exclude members of particular groups	
**Lack of researcher training opportunities to guide meaningful patient engagement in basic science research [R/P]**	4
Lack of research experience, preparation, and clarity around patient engagement expectations	
Enablers	Studies

**Creating a safe space where patient partners and researchers feel comfortable to collaborate [R/P]**	6
Ensure team members feel comfortable in sharing individual views	
Distribute learning materials before and after meetings	
**Consider arranging the team structure to support patient engagement [R/P]**	5
Training and resources for researchers to overcome challenges	
Critically building study team composition including an engagement coordinator	
**Develop patient engagement strategies ahead of time [R]**	2
Plan for equitable division of responsibilities to reduce the burden on the project team and help partners feel more invested	
Consider the needs of the community	
**External organizations that actively support patient engagement in basic science research projects [R/P]**	1
Enforcing and facilitating the involvement of patient partners	

Abbreviations: R and P denote themes that pertain to researchers and patient partners, respectively.

Reported enablers to patient engagement were summarized into four themes (Table 4). The most commonly reported enabler was ‘creating a safe space where patient partners and researchers felt comfortable collaborating’. Further details on items to consider when creating a safe space are outlined in Supplementary Table 4. Two additional enabling themes that should be considered when preparing to engage patients include ‘arranging the team structure to support patient engagement’, which can be achieved by designating an ‘engagement coordinator’, and ‘developing a patient engagement strategy ahead of time’; this might ultimately better ‘consider the needs of the community’ and ‘plan for equitable distribution of responsibilities’. The final enabling theme highlighted ‘external organizations that actively support patient engagement’ as an enabler.

## 4 Discussion

Our scoping review identified a number of examples of patient engagement in preclinical research that provided a rich corpus of information to facilitate future work in this field. Although the prevalence of published patient engagement initiatives in preclinical research is low, included studies collectively exhibited many examples of patient engagement across various preclinical research stages and at each level of engagement.

Of note, the majority of included studies were conducted in the United Kingdom and the United States. This may be a reflection of INVOLVE (UK based) and the Patient Centered Outcomes Research Institute (US based) being two established agencies that facilitate patient engagement in research. We identified considerations that may be unique to preclinical research. Reported challenges of patient engagement emerged from differences in research knowledge and how this might affect the quality of research and thereby potentially limit meaningful engagement. This perceived knowledge gap may be attributed to the fact that laboratory research is traditionally a non-public facing field; this may make it difficult for patient partners to fully connect with future innovations. Moreover, given the often highly technical nature of preclinical research, patient partners may not have sufficient knowledge in preclinical research to communicate effectively or, as highlighted in our review, feel comfortable sharing individual views. As a corollary, preclinical scientists may not have adequate training and/or experience to communicate with patient partners. Indeed, communication with the public (and in a non-technical manner) has only recently been highlighted as a needed skill in basic science [50].

Bridging the gap in a non-patient or non-public facing area of research is not easily accomplished and requires special considerations. A common theme that emerged from our analysis was the important role played by patient organizations or engagement coordinators in supporting collaboration between preclinical researchers and patient partners. This role would represent a connection between both groups that understands the perspectives, strengths, and weaknesses of each. Personnel in this role may initiate connections between researchers and the public by identifying patients that are interested in becoming partners and dissolving barriers.

As highlighted in this review, interested patients and members of patient organizations are being invited to attend basic science conferences. This preliminary step to patient engagement may be an effective strategy to begin bridging the gap between patients and basic scientists. Considerations and methods to engage patients within scientific conferences have been studied in the clinical research field including offering accommodations to encourage attendance, extending the invitation to diverse groups, co-developing conference activities, and co-evaluating the patient centeredness of presentations or abstracts [51]. Although invitations to conferences may initially bridge the gap between the public and basic science, our review has captured activities that may engage patient partners throughout individual basic science research project (from education and priority setting to dissemination). Certainly, future efforts to engage patient partners should consider lessons learned captured by our review including recognizing patient partner contributions, maintaining diversity, and emphasizing collaboration.

Patient engagement in clinical research has been described in a number of systematic reviews [52], [53], [54], and the impacts of these activities are under empirical investigation [55,56]. Despite the relative paucity of work relating to patient engagement in preclinical research, our findings echo reported outcomes of patient engagement in clinical research. A recent scoping review [53] of patient engagement initiatives in health research highlighted similar benefits of engagement, including improving communication with the public and creating mutual learning opportunities that build new skills and generate fresh perspectives. More specifically, we identified patient engagement as a way for researchers to recognize the real-life implications of their work and for patient partners to learn about preclinical research being conducted in a field that is of interest to them. Both opportunities are unlikely to occur without a partnership between preclinical researchers and patient partners.

Although many parallels have been drawn from patient engagement in clinical research, it is important to highlight where engagement differs. Our review did not identify examples of patient engagement reported at the data collection and data analysis stages. While this may not be surprising, patient engagement is beginning to emerge in clinical data collection processes which highlights a unique consideration [55]. One major difference between preclinical and clinical research is that the conduct of preclinical research in laboratories is not patient facing. Thus, the research environment may contribute to the lack of patient engagement in certain preclinical data domains given limitations around routine access for patients to animal lab facilities.

Our results suggest that patient engagement is feasible in preclinical laboratory research. Although this review highlights the current state of a novel area of patient engagement, a few limitations and potential areas of bias should be noted. Firstly, the individual included studies often provided rich amounts of information; however, some pertinent, granular details may not have been captured in our thematic analysis; we recommend that preclinical researchers interested in patient engagement should review the included studies for further details. Our synthesis may have been impacted by the interests of the researchers of the included articles. Since most included articles were written from the perspective of the researcher, the reported impacts of patient engagement may not fully reflect the views of patient partners. In addition, unclear reporting of individual studies introduces potential risk of bias in this review. For example, several patient engagement activities, including participation in funding panels or committee work, may not be published and thus not picked up by systematic database searches. Alternatively, our findings may be influenced by the lack of published null or negative preclinical research. Despite our use of a comprehensive search strategy (designed in collaboration with an information specialist), there are likely other examples of patient engagement in preclinical research that we did not capture since different terms and definitions are being used interchangeably in the literature (i.e. engagement, involvement, empowerment, patient-centred research) [54]. Finally, some journals do not accept patient partners as authors [57] which may explain why few studies recognized patient partner contribution through co-authorship.

Our review provides a current summary of patient engagement in preclinical laboratory research. In order to accelerate development of this area, patient organizations, funding agencies, and research institutions should consider how to facilitate opportunities for preclinical laboratory researchers and patients to interact. Future research should focus on proper reporting of patient engagement activities in preclinical laboratory research and assessment of the impacts of such practices on research conduct (Supplementary Table 5). Indeed, members of our group will be using results of this review to generate an evidence informed framework to help guide the development and conduct of future patient engagement in preclinical research.

## Declaration of Competing Interest

All authors have completed the ICMJE uniform disclosure form at www.icmje.org/coi_disclosure.pdf and declare: no support from any organisation for the submitted work; no financial relationships with any organisations that might have an interest in the submitted work in the previous three years; no other relationships or activities that could appear to have influenced the submitted work. DPR reports other relationships and activities from Institute of Musculoskeletal Health and Arthritis (CIHR), personal fees from Janssen, other from Various pharmaceutical companies, other from Clinical Trials Ontario, other from Chronic Pain Network, outside the submitted work; DPR is a scientist by training and lives with rheumatoid arthritis.

## Appendix Supplementary materials

Image, application 1

Image, application 2

Image, application 3
