# Supplementary material for: Patient engagement in preclinical laboratory research: A scoping review
Source: eBioMedicine. 2021 Jul 17;70:103484. doi: 10.1016/j.ebiom.2021.103484 (PMC8318845; doi:10.1016/j.ebiom.2021.103484)

**Appendix 1. PRISMA-ScR Checklist**

| **SECTION** | **ITEM** | **PRISMA-ScR CHECKLIST ITEM** | **REPORTED ON PAGE #** |
| --- | --- | --- | --- |
| **TITLE** | | | |
| Title | 1 | Identify the report as a scoping review. | 1 |
| **ABSTRACT** | | | |
| Structured summary | 2 | Provide a structured summary that includes (as applicable): background, objectives, eligibility criteria, sources of evidence, charting methods, results, and conclusions that relate to the review questions and objectives. | 2 |
| **INTRODUCTION** | | | |
| Rationale | 3 | Describe the rationale for the review in the context of what is already known. Explain why the review questions/objectives lend themselves to a scoping review approach. | 4 |
| Objectives | 4 | Provide an explicit statement of the questions and objectives being addressed with reference to their key elements (e.g., population or participants, concepts, and context) or other relevant key elements used to conceptualize the review questions and/or objectives. | 5 |
| **METHODS** | | | |
| Protocol and registration | 5 | Indicate whether a review protocol exists; state if and where it can be accessed (e.g., a Web address); and if available, provide registration information, including the registration number. | 6 |
| Eligibility criteria | 6 | Specify characteristics of the sources of evidence used as eligibility criteria (e.g., years considered, language, and publication status), and provide a rationale. | 7-8 |
| Information sources* | 7 | Describe all information sources in the search (e.g., databases with dates of coverage and contact with authors to identify additional sources), as well as the date the most recent search was executed. | 6-7 |
| Search | 8 | Present the full electronic search strategy for at least 1 database, including any limits used, such that it could be repeated. | 40-42 |
| Selection of sources of evidence† | 9 | State the process for selecting sources of evidence (i.e., screening and eligibility) included in the scoping review. | 7-8 |
| Data charting process‡ | 10 | Describe the methods of charting data from the included sources of evidence (e.g., calibrated forms or forms that have been tested by the team before their use, and whether data charting was done independently or in duplicate) and any processes for obtaining and confirming data from investigators. | 8-9 |
| Data items | 11 | List and define all variables for which data were sought and any assumptions and simplifications made. | 43 |
| Critical appraisal of individual sources of evidence§ | 12 | If done, provide a rationale for conducting a critical appraisal of included sources of evidence; describe the methods used and how this information was used in any data synthesis (if appropriate). | Not applicable |
| Synthesis of results | 13 | Describe the methods of handling and summarizing the data that were charted. | 8-9 |

| **SECTION** | **ITEM** | **PRISMA-ScR CHECKLIST ITEM** | **REPORTED ON PAGE #** |
| --- | --- | --- | --- |
| **RESULTS** | | | |
| Selection of sources of evidence | 14 | Give numbers of sources of evidence screened, assessed for eligibility, and included in the review, with reasons for exclusions at each stage, ideally using a flow diagram. | 10 |
| Characteristics of sources of evidence | 15 | For each source of evidence, present characteristics for which data were charted and provide the citations. | 12-14 |
| Critical appraisal within sources of evidence | 16 | If done, present data on critical appraisal of included sources of evidence (see item 12). | Not applicable |
| Results of individual sources of evidence | 17 | For each included source of evidence, present the relevant data that were charted that relate to the review questions and objectives. | 10-26 |
| Synthesis of results | 18 | Summarize and/or present the charting results as they relate to the review questions and objectives. | 10-26 |
| **DISCUSSION** | | | |
| Summary of evidence | 19 | Summarize the main results (including an overview of concepts, themes, and types of evidence available), link to the review questions and objectives, and consider the relevance to key groups. | 27 |
| Limitations | 20 | Discuss the limitations of the scoping review process. | 29-30 |
| Conclusions | 21 | Provide a general interpretation of the results with respect to the review questions and objectives, as well as potential implications and/or next steps. | 30 |
| **FUNDING** | | | |
| Funding | 22 | Describe sources of funding for the included sources of evidence, as well as sources of funding for the scoping review. Describe the role of the funders of the scoping review. | 9-10 |

JBI = Joanna Briggs Institute; PRISMA-ScR = Preferred Reporting Items for Systematic reviews and Meta-Analyses extension for Scoping Reviews.

* Where *sources of evidence* (see second footnote) are compiled from, such as bibliographic databases, social media platforms, and Web sites.

† A more inclusive/heterogeneous term used to account for the different types of evidence or data sources (e.g., quantitative and/or qualitative research, expert opinion, and policy documents) that may be eligible in a scoping review as opposed to only studies. This is not to be confused with *information sources* (see first footnote).

‡ The frameworks by Arksey and O’Malley (6) and Levac and colleagues (7) and the JBI guidance (4, 5) refer to the process of data extraction in a scoping review as data charting*.*

§ The process of systematically examining research evidence to assess its validity, results, and relevance before using it to inform a decision. This term is used for items 12 and 19 instead of "risk of bias" (which is more applicable to systematic reviews of interventions) to include and acknowledge the various sources of evidence that may be used in a scoping review (e.g., quantitative and/or qualitative research, expert opinion, and policy document).

*From:* Tricco AC, Lillie E, Zarin W, O'Brien KK, Colquhoun H, Levac D, et al. PRISMA Extension for Scoping Reviews (PRISMAScR): Checklist and Explanation. Ann Intern Med. 2018;169:467–473. [doi: 10.7326/M18-0850](http://annals.org/aim/fullarticle/2700389/prisma-extension-scoping-reviews-prisma-scr-checklist-explanation).

**Appendix 2. GRIPP2 Checklist short form**

| **1: Aim-Report the aim of PPI in the study** | The aim of involving patient partners is to ensure study design (i.e. identification of the research question, research outcomes, content analysis, dissemination of research findings) and conduct are influenced by the patient and caregiver perspectives. |
| --- | --- |
| **2: Methods-Provide a clear description of the methods used for PPI in the study** | Patient partners attended bi-monthly videoconferences, which allowed for regular discussion between all team members regardless of their place of residence. |
| **3: Study results—Report the results of PPI in the study, including both positive and negative outcomes** | Patient partners reviewed our scoping review protocol, provided suggestions for data elements to be extracted, and also participated in several discussions as we identified emerging themes from the content analysis. |
| **4. Discussion and conclusions—Comment on the extent to which PPI influenced the study overall. Describe positive and negative effects** | Overall, patient partners positively impacted study conduct. For example, our patient partners guided our strategy of using the IAP2 spectrum to capture the level of engagement and confirmed that our categorization was appropriate. |
| **Reflections/critical perspective-Comment critically on the study, reflecting on the things that went well and those that did not, so others can learn from this experience** | When patient partners were unavailable to attend bi-monthly team meetings, research assistants offered to meet individually and relay discussion points and elicit feedback. This ensured that all team members were regularly updated on project progress despite scheduling conflicts. Patient partners were compensated through honorariums and this was discussed at study onset. The SPOR Evidence Alliance Patient Partner Appreciation Policy and Protocol was used as guidance for rates of compensation (58). In retrospect, we should have made more of an effort to ensure all members felt comfortable sharing their individual views. |

Staniszewska S, Brett J, Simera I, et al. GRIPP2 reporting checklists: tools to improve reporting of patient and public involvement in research. *BMJ* 2017;358:j3453. doi: 10.1136/bmj.j3453

Our patient partners, Dawn Richards and Kathryn Hendrick, currently reside in Toronto, Canada. Dawn is the founder of Five02 Labs Inc., and provides patient and scientific services to clients, one of which is Clinical Trials Ontario where she is the Director of Patient and Public Engagement. Dawn lives with arthritis (rheumatoid and osteo-arthritis) and her work combines her passion for science (she has a PhD in Analytical Chemistry) with making the most of her diagnoses. Kathryn is a health care communications specialist who has experience working in various academic and research institutes across Canada. Her patient engagement journey started as an advocate for sepsis management after caring for her father who experienced repeated episodes of sepsis before his death.

**Appendix 3. Initial and updated search strategy**

Database: Embase Classic+Embase <1947 to 2019 June 21>, Ovid MEDLINE(R) ALL <1946 to June 21, 2019>

Search Strategy:

--------------------------------------------------------------------------------

1     patient participation/ or consumer participation/ or patient advocacy/ or consumer advocacy/ or STAKEHOLDER PARTICIPATION/ (164209)

2     ((consumer* or patient* or stakeholder* or user* or lay or citizen* or public or client* or carer* or caregiver* or surrogate* or family) adj2 (particip* or involv* or represent* or collaborat* or consult* or contribut* or engag* or conduct*)).tw. (393259)

3     (citizen* adj (council? or jury or juries or panel?)).tw. (288)

4     (public adj (meeting? or forum? or consultation*)).tw. or public meeting*.kw. or public forum*.kw. or public consultation*.kw. (1731)

5     (patient* particip* or patient* involv* or patient represent* or patient* collaborat* or patient* consult* or patient* contrib* or patient* engag* or patient* deliberat* or patient* dialogue or patient* opinion* or patient* council* or patient* committee* or patient* partner* or patient voice*).kw. (4430)

6     (patient adj4 engag*).tw. (9562)

7     or/1-6 (543099)

8     ((preclinic* or pre clinic*) adj2 (trial* or research* or stud* or experiment*)).tw. (73449)

9     Translational Medical Research/ (21059)

10     (translation* adj2 research).tw. (24880)

11     (translation* research* or translation* medical research*).kw. (4016)

12     (preclinic* or pre clinic*).kw,ti. (45598)

13     (basic adj3 (science* or research)).tw. (69969)

14     Animal Experimentation/ (2391698)

15     ((lab or laborator*) adj1 (trial* or research* or stud* or experiment*)).tw. (98649)

16     ((lab based or laborator* based) adj1 (trial* or research* or stud* or experiment*)).tw. (2842)

17     (animal research or animal experiment* or animal testing).tw,kw. (63710)

18     (Genomic* Research or gene research or genes research or genetic research).tw,kw. (13892)

19     *genetic research/ or *human genome project/ (81195)

20     Genomics/mt or *genomics/ (47024)

21     exp *animals/ and (*research/ or *biomedical research/) (31452)

22     (drug evaluation, preclinical/ or drug discovery/) and early.tw,kw. (9290)

23     ((early drug* or early medicine*) adj3 (research or development)).tw. (995)

24     or/8-23 (2891529)

25     7 and 24 (7445)

26     25 use medall (1973)

27     conference abstract.pt. or abstract.so. (3441430)

28     patient participation/ (49471)

29     stakeholder engagement/ (1694)

30     patient advocacy/ (44777)

31     consumer advocacy/ (6453)

32     ((consumer* or patient* or stakeholder* or user* or lay or citizen* or public or client* or carer* or caregiver* or surrogate* or family) adj2 (particip* or involv* or represent* or collaborat* or consult* or contribut* or engag* or conduct*)).tw. (393259)

33     (citizen* adj (council? or jury or juries or panel?)).tw. (288)

34     (public adj (meeting? or forum? or consultation*)).tw. (1704)

35     or/28-34 (481060)

36     preclinical study/ or *animal experiment/ (30409)

37     ((preclinic* or pre clinic*) adj2 (trial* or research* or stud* or experiment*)).tw. (73449)

38     translational research/ (25312)

39     (translation* adj2 research).tw. (24880)

40     (preclinic* or pre clinic*).ti. (40867)

41     (basic adj3 (science* or research)).tw. (69969)

42     ((lab or laborator*) adj1 (trial* or research* or stud* or experiment*)).tw. (98649)

43     ((lab based or laborator* based) adj1 (trial* or research* or stud* or experiment*)).tw. (2842)

44     (animal research or animal experiment* or animal testing).tw. (62207)

45     (Genomic* Research or gene research or genes research or genetic research).tw. (13669)

46     *genetics/ or *human genome project/ (102805)

47     *genomics/ (41917)

48     *drug development/ and early.tw. (918)

49     ((early drug* or early medicine*) adj3 (research or development)).tw. (995)

50     or/36-49 (539413)

51     35 and 50 (4643)

52     conference abstract.pt. or abstract.so. (3441430)

53     51 not 52 (3845)

54     53 use emczd (2141)

55     26 or 54 (4114)

56     remove duplicates from 55 (2759)

**57     56 use medall (1971)  Medline**

**58     56 use emczd (788)     Embase**

Database: Embase Classic+Embase <1947 to 2021 April 05> , Ovid MEDLINE(R) ALL <1946 to April 05, 2021>

Search Strategy:

--------------------------------------------------------------------------------

1     patient participation/ or consumer participation/ or patient advocacy/ or consumer advocacy/ or STAKEHOLDER PARTICIPATION/ (181734)

2     ((consumer* or patient* or stakeholder* or user* or lay or citizen* or public or client* or carer* or caregiver* or surrogate* or family) adj2 (particip* or involv* or represent* or collaborat* or consult* or contribut* or engag* or conduct*)).tw. (466163)

3     (citizen* adj (council? or jury or juries or panel?)).tw. (337)

4     (public adj (meeting? or forum? or consultation*)).tw. or public meeting*.kw. or public forum*.kw. or public consultation*.kw. (2008)

5     (patient* particip* or patient* involv* or patient represent* or patient* collaborat* or patient* consult* or patient* contrib* or patient* engag* or patient* deliberat* or patient* dialogue or patient* opinion* or patient* council* or patient* committee* or patient* partner* or patient voice*).kw. (6363)

6     (patient adj4 engag*).tw. (13172)

7     or/1-6 (630167)

8     ((preclinic* or pre clinic*) adj2 (trial* or research* or stud* or experiment*)).tw. (89649)

9     Translational Medical Research/ (23847)

10     (translation* adj2 research).tw. (29927)

11     (translation* research* or translation* medical research*).kw. (5397)

12     (preclinic* or pre clinic*).kw,ti. (55614)

13     (basic adj3 (science* or research)).tw. (79049)

14     Animal Experimentation/ (2690704)

15     ((lab or laborator*) adj1 (trial* or research* or stud* or experiment*)).tw. (109633)

16     ((lab based or laborator* based) adj1 (trial* or research* or stud* or experiment*)).tw. (3349)

17     (animal research or animal experiment* or animal testing).tw,kw. (70472)

18     (Genomic* Research or gene research or genes research or genetic research).tw,kw. (15787)

19     *genetic research/ or *human genome project/ (105980)

20     Genomics/mt or *genomics/ (31959)

21     exp *animals/ and (*research/ or *biomedical research/) (31713)

22     (drug evaluation, preclinical/ or drug discovery/) and early.tw,kw. (11734)

23     ((early drug* or early medicine*) adj3 (research or development)).tw. (1149)

24     or/8-23 (3251732)

25     7 and 24 (9006)

26     25 use medall (2391)

27     ("20190618" or "20190619" or 2019062* or 2019063* or 201907* or 201908* or 201909* or 20191* or 2020* or 2021*).dt. (2592416)

**28     26 and 27 (361)  Medline**

29     patient participation/ (55935)

30     stakeholder engagement/ (4886)

31     patient advocacy/ (46253)

32     consumer advocacy/ (6691)

33     ((consumer* or patient* or stakeholder* or user* or lay or citizen* or public or client* or carer* or caregiver* or surrogate* or family) adj2 (particip* or involv* or represent* or collaborat* or consult* or contribut* or engag* or conduct*)).tw. (466163)

34     (citizen* adj (council? or jury or juries or panel?)).tw. (337)

35     (public adj (meeting? or forum? or consultation*)).tw. (1979)

36     or/29-35 (561325)

37     preclinical study/ or *animal experiment/ (45975)

38     ((preclinic* or pre clinic*) adj2 (trial* or research* or stud* or experiment*)).tw. (89649)

39     translational research/ (30201)

40     (translation* adj2 research).tw. (29927)

41     (preclinic* or pre clinic*).ti. (49076)

42     (basic adj3 (science* or research)).tw. (79049)

43     ((lab or laborator*) adj1 (trial* or research* or stud* or experiment*)).tw. (109633)

44     ((lab based or laborator* based) adj1 (trial* or research* or stud* or experiment*)).tw. (3349)

45     (animal research or animal experiment* or animal testing).tw. (68615)

46     (Genomic* Research or gene research or genes research or genetic research).tw. (15498)

47     *genetics/ or *human genome project/ (128371)

48     *genomics/ (49267)

49     *drug development/ and early.tw. (1196)

50     ((early drug* or early medicine*) adj3 (research or development)).tw. (1149)

51     or/37-50 (636965)

52     36 and 51 (5743)

53     conference abstract.pt. or abstract.so. (4072248)

54     52 not 53 (4701)

55     54 use emczd (2630)

56     ("20190618" or "20190619" or 2019062* or 2019063* or 201907* or 201908* or 201909* or 20191* or 2020* or 2021*).dc. (3820999)

**57     55 and 56 (543)  Embase**

58     28 or 57 (904)

59     remove duplicates from 58 (644)

60     59 use medall (358)  Medline

61     59 use emczd (286)  Embase

**Appendix 4. A full list of extracted data items**

| Domain | Question |
| --- | --- |
| Study Characteristics | 1. Study author 2. Year of publication 3. Country of corresponding author 4. Journal/source of publication 5. Funding received |
| Research Characteristics | 1. Area of research (i.e. cardiac, cancer, neurological, etc.) 2. Type of research (in vitro, in vivo, other, etc.) |
| Patient Engagement Strategy Characteristics | 1. Stated reason(s) to engage patients as partners (e.g. theoretical rationale, influences) 2. Type(s) of stakeholders engaged (e.g. patient, caregiver, family member) 3. Patient partner characteristics (i.e. educational background) 4. Method of patient partner recruitment 5. Number of patients engaged 6. Stage(s) of research at time(s) of patient engagement 7. Level of patient partner engagement [defined by International Association for Public Participation (IAP2)](14) 8. Length of engagement (e.g. once, more than once, amount of time) 9. Terminology used to describe patient engagement 10. Type and extent of training patients received 11. Type and extent of training researchers received to engage patient partners 12. Credit given to patients (i.e. authorship, acknowledgement, etc.) 13. Whether patients were compensated or reimbursed |
| Outcomes & Lessons from Patient Engagement | 1. Economic assessment/costs of patient engagement 2. Contextual and process factors (barriers and enablers from the perspectives of both patient partners and researchers) 3. Measured or stated impact of patient engagement 4. Reported benefits, disadvantages or lessons learned (from the perspective of both patient partners and researchers) |

**Supplementary Table 1. Terminology used to describe patient engagement and reported patient partner characteristics (n=30)**

| **Study details** | **Terminology used to describe patient engagement** | **Patient partner characteristics** | | | | | |
| --- | --- | --- | --- | --- | --- | --- | --- |
|  |  | Age | Gender | Country of residence/  ethnicity | Language | Educational background | Profession |
| Rheault, 2020 (18) | Patient engagement | Teenagers | N/R | Australia, China, France, Germany, Italy, Netherlands, Spain, United Kingdome and the United States of America | N/R | N/R | N/R |
| van den Berg, 2019 (19) | N/R | N/R | N/R | N/R | N/R | N/R | N/R |
| Boenink, 2018 (20) | Patient involvement | N/R | N/R | N/R | N/R | N/R | N/R |
| Russell, 2018 (21) | Patient and public involvement | N/R | N/R | N/R | N/R | N/R | N/R |
| Tamagnini, 2018 (22) | Patient and public involvement, public engagement | N/R | N/R | N/R | N/R | One patient partner had a strong scientific background | One patient partner was a retired Royal Navy officer |
| Frazier, 2018 (23) | Community engagement | N/R | N/R | N/R | N/R | N/R | N/R |
| McDonnell, 2018 (25) | Patient and public involvement (PPI), patient and public engagement | N/R | N/R | N/R | N/R | N/R | N/R |
| Parsons, 2017 (26) | Young people involvement, patient and public involvement (PPI) | Young people aged 11-24, mean age 16 years old | 18 males and 45 females | N/R | English speaking | N/R | N/R |
| Zoeller, 2014 (27) | Public participation | Participants aged 19-82 | 1 male and 4 females | Germany, Ireland, Sweden | N/R | 5 participants have academic qualifications/degrees (two University and 3 technical college) | Authors reported profession of each participant |
| Filocamo, 2013 (28) | Not specified | N/R | N/R | N/R | N/R | N/R | N/R |
| Black, 2013 (29) | Engagement | N/R | N/R | N/R | N/R | N/R | N/R |
| Godard, 2007 (30) | Community engagement | Participants aged 25-49 and 50-74 | N/R | Participants were Arab, Ashkenazi, English, French, Greek, Hispanic and Italian living in various regions of Quebec | French and English speaking | N/R | Working, middle, and upper class |
| *Haga, 2013 (31) | Public engagement | Middle school students (12-13 years old) and parents/guardians | N/R | 53% of parents/  guardians were African American and 43% white | N/R | 80.8% of parents/guardians had a Bachelors’ or graduate degree | 62% of parents/guardians reported having a household income of more than $80,000 |
| *O’Daniel, 2012 (32) | Participant engagement | Middle school students (12-13 years old) and parents/guardians | N/R | 53% of parents/  guardians were African American and 43% white | N/R | 80.8% of parents/guardians had a Bachelors’ or graduate degree | 62% of parents/guardians reported having a household income of more than $80,000 |
| Terry, 2012 (33) | Community engagement | Focus group participants aged 18-102 | Focus groups included 8 males and 18 females | 4 members self-identified as white, 10 as African American, and 15 as Latino | 9 participants spoke Spanish as a first language | High school graduates (n=5), some college education (n=7), college graduates (n=10), and graduate school (n=3) | Self-reported income level of <$15,000 (n=7), $15,000-$30,000 (n=4), $30,000-$45,000 (n=5), $45,000-$60,000(n=1), and $60,000+ (n=8) |
| Pulver, 2011 (34) | Public engagement | Children 0-18 and adults 19+ | N/R | N/R | N/R | N/R | N/R |
| Arturi, 2011 (35) | Not specified | N/R | N/R | N/R | N/R | N/R | Both founders have robust business careers |
| Baart, 2011 (36) | Patient involvement, patient and family member involvement, action research | N/R | N/R | N/R | N/R | N/R | N/R |
| Boon, 2010 (37) | Involvement | N/R | N/R | N/R | N/R | N/R | N/R |
| Van Olphen, 2009 (38) | Community-based participatory research (CBPR) | N/R | N/R | N/R | N/R | N/R | N/R |
| Haddow, 2008 (39) | Public engagement | N/R | N/R | Scotland | N/R | N/R | N/R |
| Riter, 2019 (40) | Public engagement, “community members” to encompass patients, survivors, family members, and public | N/R | N/R | N/R | N/R | N/R | N/R |
| Mollan, 2019 (42) | Patient and public involvement (PPI) | Median age 35 | 96% of survey respondents were female | 92% of survey respondents were White, 3% were Black or Asian, and 4% were of multiple ethnic backgrounds | N/R | N/R | N/R |
| Costello, 2020 (43) | Patient and public involvement (PPI) | Participants aged 10-20 | Overall attendee sex ratio was 4:11 male to female | N/R | N/R | N/R | N/R |
| Davies, 2021 (45) | Patient and public involvement (PPI), patient and public engagement | N/R | N/R | N/R | N/R | N/R | N/R |
| Taruscio, 2020 (46) | Patient involvement, patient engagement | N/R | N/R | N/R | N/R | N/R | N/R |
| Moore, 2020 (47) | Patient and public involvement (PPI) | N/R | 2 males, 2 females | N/R | N/R | N/R | N/R |
| Mahler, 2020 (48) | Public engagement | N/R | N/R | N/R | N/R | N/R | N/R |
| Birch, 2020 (49) | Patient and public involvement (PPI) | Two were aged between 30-39, two aged 50-59, and two aged 60-69. | 8 females | Estonia (1), Germany (1), Netherlands (1), Sweden (1), Romania (2), United Kingdom (3). | N/R | N/R | N/R |

*Denotes articles describing the same study.

Abbreviations: N/R = Not reported

**Supplementary Table 2. Credit given to patient partners (n=30).**

| **Study Details** | **Credit given to patient partners**** | | | | | **Financial compensation of patient partners***** | | | | |
| --- | --- | --- | --- | --- | --- | --- | --- | --- | --- | --- |
|  | Acknowledgement | Authorship | Gift | Listed as a co-investigator | N/R | Compensated | Reimbursed | Received a gift | Not applicable | N/R |
| Rheault, 2020 (18) |  |  |  |  | 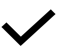 |  |  |  |  | 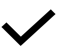 |
| van den Berg, 2019 (19) | 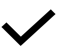 |  |  |  |  |  |  |  |  | 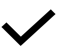 |
| Boenink, 2018 (20) | 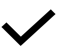 |  |  |  |  |  |  |  |  | 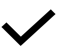 |
| Russell, 2018 (21) |  |  |  |  | 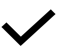 |  |  |  |  | 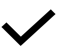 |
| Tamagnini, 2018 (22) |  | 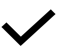 |  |  |  |  |  |  |  | 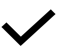 |
| Frazier, 2018 (23) | 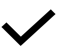 |  |  |  |  |  |  |  |  | 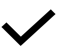 |
| Talebizadeh, 2018 (24) | 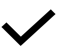 |  |  |  |  |  |  |  |  | 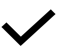 |
| McDonnell, 2018 (25) | 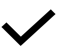 |  |  |  |  |  |  |  |  | 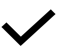 |
| Parsons, 2017 (26) | 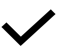 |  |  |  |  | 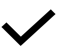 |  | 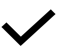 |  |  |
| Zoeller, 2014 (27) |  |  |  |  | 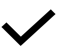 |  |  |  |  | 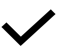 |
| Filocamo, 2013 (28) |  |  |  |  | 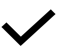 |  |  |  |  | 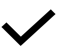 |
| Black, 2013 (29) |  | 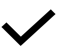 |  |  |  |  |  |  | 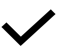 |  |
| Godard, 2007 (30) |  |  |  |  | 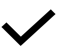 |  |  |  |  | 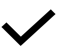 |
| *Haga, 2013 (31) |  |  |  |  | 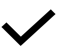 |  |  |  |  | 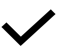 |
| *O’Daniel, 2012 (32) |  |  | 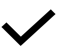 |  |  |  |  |  |  | 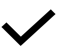 |
| Terry, 2012 (33) | 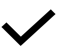 |  |  |  |  | 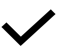 | 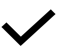 |  |  |  |
| Pulver, 2011 (34) |  |  |  |  | 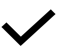 |  |  |  |  | 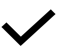 |
| Arturi, 2011 (35) |  | 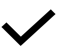 |  |  | 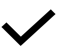 |  |  |  | 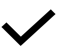 |  |
| Baart, 2011 (36) |  |  |  |  | 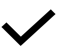 |  |  |  |  | 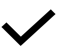 |
| Boon, 2010 (37) |  |  |  |  | 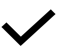 |  |  |  | 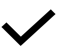 |  |
| Van Olphen, 2009 (38) |  | 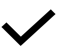 |  | 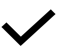 | 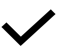 |  |  |  |  | 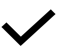 |
| Haddow, 2008 (39) |  |  |  |  | 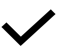 |  |  |  |  | 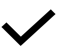 |
| Riter, 2019 (40) |  |  | 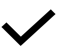 |  | 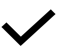 |  |  |  |  | 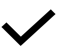 |
| Mollan, 2019 (42) |  |  |  |  | 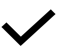 |  |  |  |  | 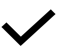 |
| Costello, 2020 (43) | 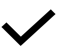 |  |  |  |  |  |  |  |  | 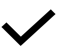 |
| Davies, 2021 (45) |  |  |  |  | 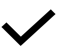 |  |  |  |  | 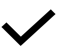 |
| Taruscio, 2020 (46) | 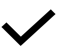 |  |  |  |  |  |  |  |  | 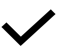 |
| Moore, 2020 (47) | 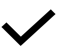 |  |  |  |  |  |  |  |  | 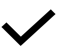 |
| Mahler, 2020 (48) |  |  |  |  | 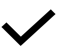 |  |  |  |  | 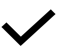 |
| Birch, 2020 (49) | 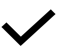 | 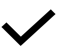 |  |  |  |  | 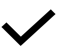 |  |  |  |
| **Total**  **(%)****** | **11**  **(38)** | **5**  **(17)** | **2 (7)** | **1**  **(3)** | **16 (55)** | **2**  **(7)** | **2**  **(7)** | **1**  **(3)** | **3**  **(10)** | **23**  **(79)** |

*Denotes articles describing the same study.

****Credit given to patient partner as a gift** – A non-monetary token of appreciation for participation in the research project.

*****Compensation of patient partner through a gift –** A method of compensating patient partners for their time outside of honoraria.

****Percentages were generated using n=29 as the denominator.

Abbreviations: N/R = Not reported

**Supplementary Table 3. Recommendations from patient engagement (n=32)**

| **Recommendations** | **Studies** |
| --- | --- |
| **Aim to recruit a diverse group of patient partners [R]** | 10 |
| Collaborating with a patient organization is an effective way of reaching and recruiting a diverse population |  |
| Consider engaging an equal number of researchers and patient partners to alleviate responsibility |  |
| **Provide educational resources to team members [R]** | 9 |
| Support team member understanding of the background and rationale for the research project |  |
| **Disagreements and frustrations should be expected [R]** | 7 |
| Acknowledge and address disagreements as they appear |  |
| **Effective and consistent communication is essential for meaningful engagement [R]** | 5 |
| Consistently communicate roles and expectations of team members |  |
| **Timing of engagement is important [R]** | 3 |
| Consider engaging patient partners from project onset to completion |  |
| **Patient partners may become engaged to different degrees [R]** | 3 |
| Consider team member capacity |  |
| **Consider the patient partner and researcher relationship as collaborative [R]** | 3 |
| Examine the effects of power relations |  |
| **Partnerships and support from patient organizations can help dissolve barriers between patients and preclinical researchers [P]** | 2 |
| For example, partnerships with patient organizations |  |
| **Researchers must recognize patient partner contributions [R]** | 1 |
| Recognition as co-authorship, compensation, acknowledgement etc. |  |
| **It is crucial to evaluate the impacts of patient engagement [R]** | 1 |
| Evaluation will improve future initiatives and bi-directional knowledge translation |  |

Abbreviations: R and P denote themes that pertain to researchers and patient partners, respectively.

**Supplementary Table 4. Items for researchers to consider when creating a space for open conversation (20, 22, 36)**

| - Do not expect patient partners to have prior scientific knowledge or to speak in scientific language - Keep meeting agendas open for patient partners to contribute discussion topics and circulate agendas ahead of time to allow for preparation - Present information in consideration to not overly shape patient partner’s responses - Consider using the Dialogue Model/Habermasian ideal/others(36) to effectively start patient partner-researcher interaction and help find common ground - Start the conversation about exploring appropriate methods of discussion and communication early in the engagement process to ensure a comfortable space is formed for future conversations. |
| --- |

**Supplementary Table 5. Suggested future directions for research initiatives focused on patient engagement in preclinical laboratory research**

| Future directions |
| --- |
| - Develop and assess resources to support both researchers and patient partners throughout meaningful patient engagement in preclinical research - Assess the impact of engagement in laboratory research - Exploring patient engagement activities at the data collection and data analysis stages of laboratory research - Identifying patient partner and preclinical researcher barriers and incentives to engagement - Development of reporting guidelines for patient engagement in preclinical laboratory research |

**Supplementary Figure 1. Geographical distribution of included articles (n=32).**


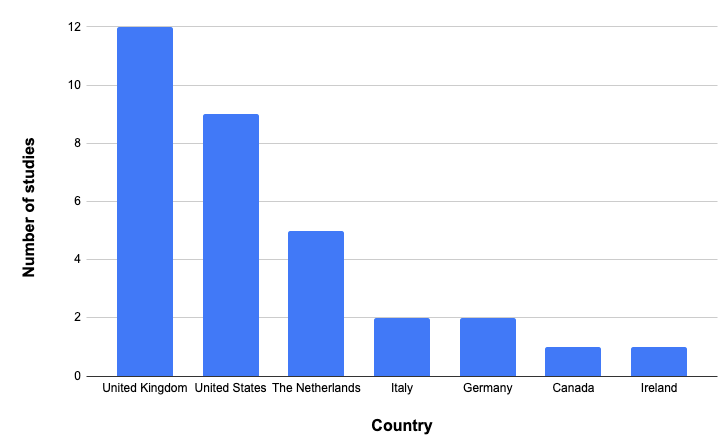


**Supplementary Figure 2. Amended International Association of Public Participation spectrum.(14)**


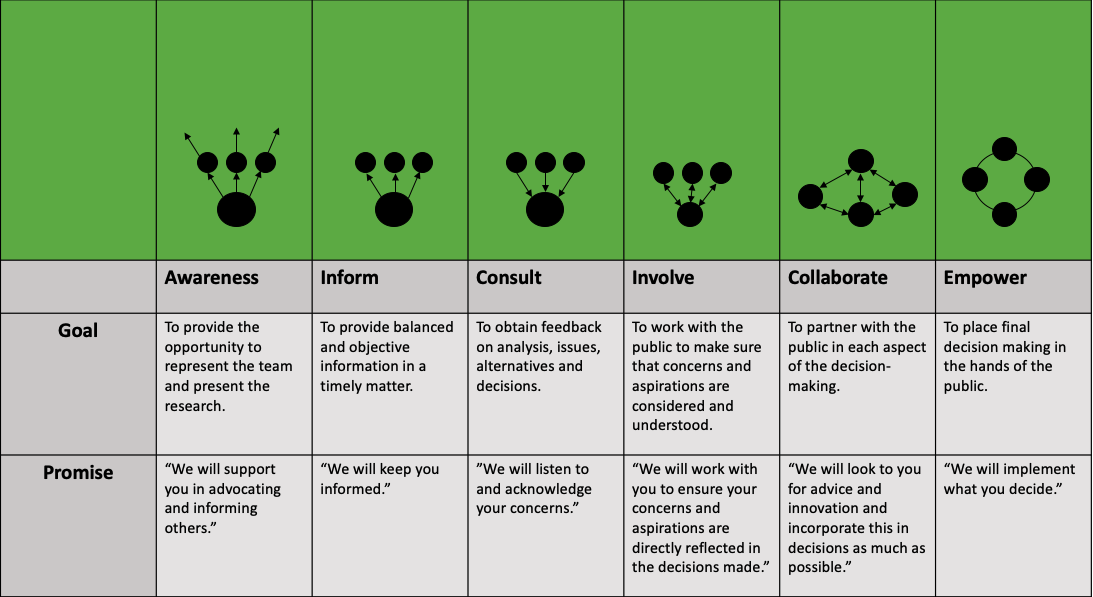

Supplement: Supplementary file 1 [file mmc1.docx]
