# Supplementary figures and images for: Patient engagement in preclinical laboratory research: A scoping review
Source: eBioMedicine. 2021 Jul 17;70:103484. doi: 10.1016/j.ebiom.2021.103484 (PMC8318845; doi:10.1016/j.ebiom.2021.103484)

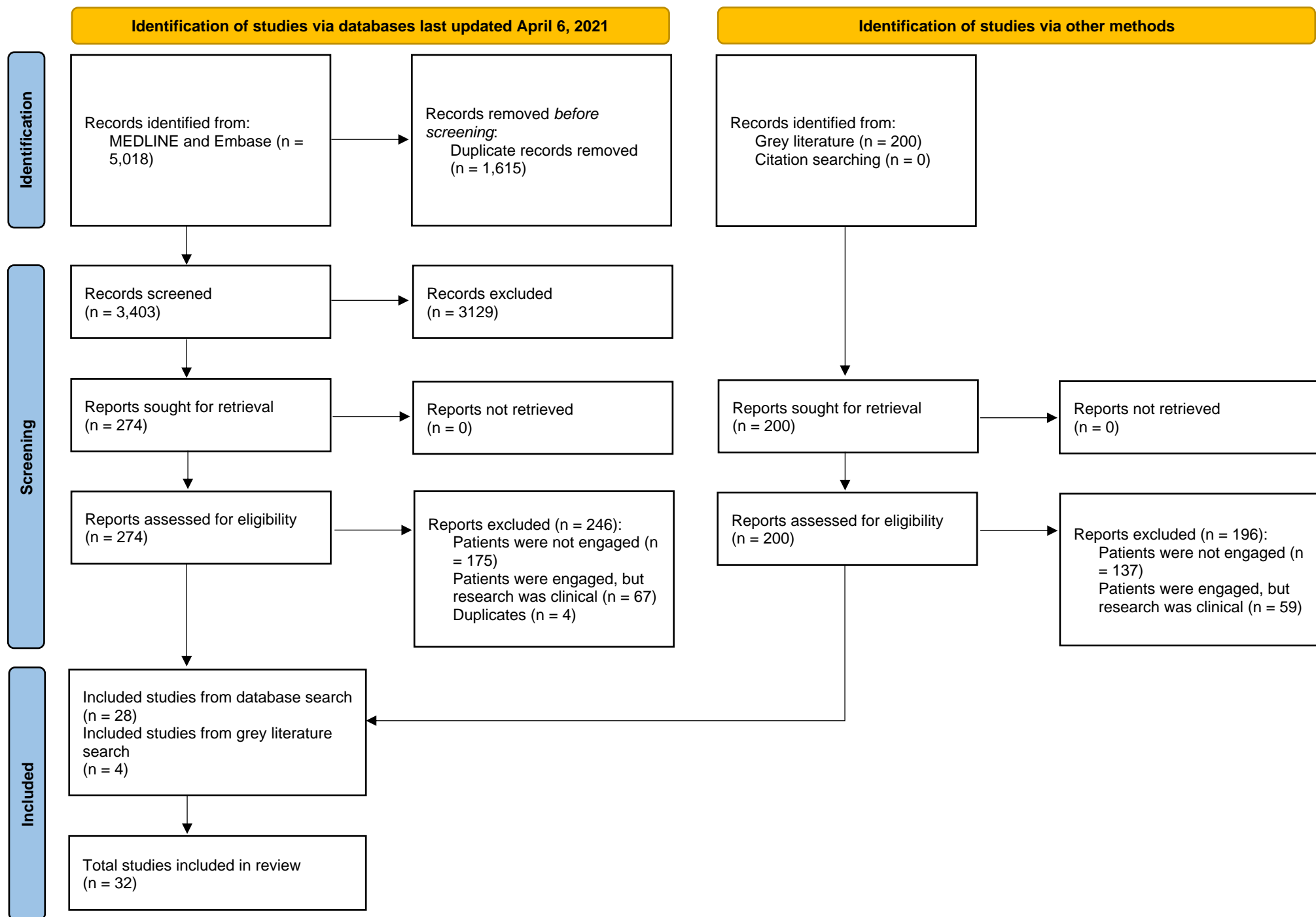

Supplement: Supplementary file 3 [file mmc3.pdf]
